# Changes in Serum MMP-9 and TIMP-1 Concentrations in Children with Inflammatory Bowel Disease over a Six-Month Observation Period

**DOI:** 10.3390/ijms27209162

**Published:** 2026-10-15

**Authors:** Katarzyna Zdanowicz, Dariusz Marek Lebensztejn, Urszula Daniluk

**Affiliations:** Department of Pediatrics, Gastroenterology, Hepatology, Nutrition, Allergology and Pulmonology, Medical University of Bialystok, Waszyngtona 17, 15-274 Bialystok, Poland; dariusz.lebensztejn@umb.edu.pl (D.M.L.); urszula.daniluk@umb.edu.pl (U.D.)

**Keywords:** Crohn’s disease, ulcerative colitis, children, MMP-9, TIMP-1, biomarkers

## Abstract

Inflammatory bowel disease (IBD) is a chronic condition with a long-term and often unpredictable course. The aim of our study was to evaluate serum levels of matrix metalloproteinase-9 (MMP-9) and tissue inhibitor of metalloproteinase-1 (TIMP-1) in children with IBD at diagnosis or disease exacerbation and during follow-up to assess their changes during the course of the disease. Patients were assessed at the time of inclusion (T0), after 3 months (T1), and after 6 months of follow-up (T2). At each time point, clinical disease activity was evaluated, along with routine laboratory tests and measurement of fecal calprotectin (fCal). Serum concentrations of MMP-9 and TIMP-1 were determined using the enzyme-linked immunosorbent assay method. Fifty-nine children with IBD were included in the study (19 with Crohn’s disease and 40 with ulcerative colitis; 39 with newly diagnosed disease and 20 with disease exacerbation). During the six-month follow-up, patients received anti-TNF-α therapy with infliximab or adalimumab; at T1, 8 patients received infliximab and 5 received adalimumab, whereas at T2, 17 patients received infliximab and 7 received adalimumab. At T0, significant differences in MMP-9 and TIMP-1 were observed between IBD patients and controls. In the IBD group, a significant decrease in MMP-9 (*p* < 0.001), TIMP-1 (*p* < 0.001), and the MMP-9/TIMP-1 ratio (*p* = 0.006) was observed over the six-month follow-up. At T1, only TIMP-1 levels differed between groups, while at T2 no significant differences were observed. Patients who achieved clinical remission showed a significant decrease in MMP-9 (*p* = 0.001) and TIMP-1 (*p* < 0.001) levels, whereas no significant changes were observed in patients who did not achieve remission. However, the percentage change in MMP-9 did not differ significantly according to clinical remission status (*p* = 0.50). Similarly, patients who achieved fCal-defined biochemical remission showed significant reductions in MMP-9 (*p* = 0.005), TIMP-1 (*p* = 0.003), and the MMP-9/TIMP-1 ratio (*p* = 0.01) over time. In patients without fCal-defined biochemical remission, MMP-9 (*p* = 0.04) and TIMP-1 (*p* = 0.01) also decreased significantly over time, whereas the MMP-9/TIMP-1 ratio did not change significantly. However, direct comparisons showed no significant differences in the percentage changes in MMP-9 (*p* = 0.28) or TIMP-1 (*p* = 0.37) according to fCal-defined biochemical remission status. In conclusion, serum MMP-9 and TIMP-1 levels change over six months in children with IBD, reflecting changes in disease activity. Changes in these biomarkers were not significantly associated with fCal-defined biochemical remission. A significant difference in TIMP-1 percentage change was observed according to clinical remission status. However, this finding should be considered exploratory and interpreted with caution.

## 1. Introduction

Inflammatory bowel disease (IBD) is a chronic, heterogeneous inflammatory disease with a multifactorial pathogenesis and an unpredictable course. It has two main subtypes: ulcerative colitis (UC) and Crohn’s disease (CD). In UC, inflammation primarily affects the mucosal and submucosal layers of the colon, whereas in CD, any part of the gastrointestinal tract can be involved, and the inflammation is transmural, with a higher frequency of fistulas and strictures [1,2]. Although UC and CD have distinct clinical and morphological features, the underlying inflammatory mechanisms appear to share common pathways, as both conditions arise from a dysregulated mucosal immune response to intestinal microbiota in genetically predisposed individuals, leading to similar patterns of chronic inflammation despite phenotypic differences [3,4,5]. The disease affects both adults and children. Notably, the course in pediatric patients is often more complex, requiring more intensive therapy and being associated with a higher risk of complications [6,7]. Therefore, there is a need to identify reliable markers that can help assess disease activity and predict its progression.

In the course of IBD, extracellular matrix remodeling plays an important role. Inflammatory processes lead to excessive activity of matrix metalloproteinases (MMPs), including MMP-9, and changes in the levels of their natural inhibitors, mainly tissue inhibitor of metalloproteinase-1 (TIMP-1) [8]. MMP-9, secreted by neutrophils, fibroblasts, and mesenchymal cells, degrades collagens and other extracellular matrix components, promoting the formation of ulcers, strictures and fistulas. It also facilitates neutrophil migration through the intestinal wall and disrupts epithelial intercellular junctions, thereby increasing intestinal permeability and contributing to tissue damage during active inflammation. Moreover, MMP-9 may serve as a marker of active inflammation, as it is predominantly produced by neutrophils and released in response to proinflammatory cytokines [9,10]. In contrast, TIMP-1, a natural inhibitor of matrix metalloproteinases, regulates MMP activity, including MMP-9, and limits excessive degradation of the extracellular matrix [11]. Collectively, these mechanisms have made MMP-9 and TIMP-1 key targets of interest in IBD research. Most studies, conducted primarily in adults, have focused on a single time point at diagnosis and the correlation of MMP-9 and TIMP-1 levels with disease severity [12,13]. However, data on their natural changes during the initial months of treatment, following diagnosis or during disease flares, are scarce in both adult and pediatric populations. Understanding these dynamics could provide valuable insights into inflammatory mechanisms and help identify potential markers for monitoring disease activity and predicting disease progression in patients.

Therefore, the purpose of our study was to evaluate the serum levels of MMP-9 and TIMP-1 in children at the time of diagnosis/exacerbation of IBD, as well as 3 and 6 months after the introduction/change of treatment. Additionally, the aim was to assess changes in MMP-9 and TIMP-1 levels in relation to the achievement of clinical remission after 6 months, fCal-defined biochemical remission at 6 months, and baseline C-reactive protein (CRP) level.

## 2. Results

### 2.1. Characteristics of the IBD Cohort

The study included 59 children with IBD: 19 with CD and 40 with UC. The patients’ ages ranged from 7 to 17 years. In both groups, patients with newly diagnosed disease predominated (63.16% for UC, 67.50% for CD). Based on the appropriate clinical scale, it was found that among children with CD the severe form was most common, whereas in UC the moderate form predominated. Demographic and clinical data are presented in Table 1.

During the six-month follow-up, patients received anti-TNF-α therapy with infliximab (IFX) or adalimumab (ADA). At T1, 8 patients received IFX and 5 received ADA, whereas at T2, 17 patients received infliximab and 7 received ADA. Patients with UC received IFX only, whereas patients with CD received either IFX or ADA according to their clinical situation.

The control group consisted of 27 children with functional abdominal pain and normal fecal calprotectin levels, without evidence of intestinal inflammation. The median age of the control group was 14 years (range, 7–17 years) and the group included 14 boys and 13 girls.

### 2.2. MMP-9, TIMP-1, and the MMP-9/TIMP-1 Ratio Measured at Study Inclusion (T0)

At T0, the study group demonstrated significantly elevated concentrations of MMP-9 and TIMP-1 compared with the control group (Table 2). Notably, the MMP-9/TIMP-1 ratio showed no statistically significant difference between these two groups. CRP was higher in children with IBD compared with controls. In the UC group at T0, higher levels of MMP-9, CRP and fCal were observed compared with the control group. However, concentrations of TIMP-1 and MMP-9/TIMP-1 ratio were comparable between the groups. Among children with CD at T0, higher levels of both MMP-9, TIMP-1 CRP and fCal were observed in the study group compared with the control group (Table 2).

In the whole IBD group, as well as in patients with UC and CD, analysis of the correlations between MMP-9, TIMP-1, and the MMP-9/TIMP-1 ratio and clinical and laboratory markers at baseline demonstrated no significant associations (Supplementary Table S1).

### 2.3. Changes in MMP-9, TIMP-1 and MMP-9/TIMP-1 Ratio During 6-Month Follow-Up

In the overall IBD cohort at T1, TIMP-1 levels were significantly higher compared with controls, whereas no differences were observed for MMP-9 (Table 2). In subgroup analysis, TIMP-1 remained significantly elevated in the CD group compared with controls at both 3 months (T1) and after 6 months (T2), while no significant differences were observed in the UC group and control group for MMP-9, TIMP-1 or the MMP-9/TIMP-1 ratio in T1 and T2. As expected, CRP differed between IBD patients and controls at T1 and T2.

We then analyzed how the concentrations of the markers changed over the course of the study. In the IBD group, a significant decrease in the concentrations of MMP-9, TIMP-1 and the MMP-9/TIMP-1 ratio was observed over six months (Table 2). In the UC group, changes were observed in MMP-9 and TIMP-1 but not in the MMP-9/TIMP-1 ratio, suggesting proportional regulation of the protease–inhibitor axis. Among the other analyzed parameters, CRP, fCal and Pediatric Ulcerative Colitis Activity Index (PUCAI) showed significant changes. In the CD group, all examined markers, including MMP-9, TIMP-1, CRP, fCal and Pediatric Crohn’s Disease Activity Index (PCDAI) showed statistically significant alterations, with the exception of the MMP-9/TIMP-1 ratio.

At the next stage of the analysis, we examined correlations between the assessed parameters (Supplementary Table S2a,b). At T1, significant correlations were identified in the IBD group between TIMP-1 and CRP (R = 0.39, *p* = 0.002). At T2, in the same group, no correlation was observed. In the UC group, only one significant correlation was identified at T2 (MMP-9/TIMP-1 ratio and fCal (R = 0.49, *p* = 0.03)). We observed the following correlation in children with CD at T1: TIMP-1 and CRP (R = 0.60, *p* = 0.007), MMP-9/TIMP-1 ratio and CRP (R = −0.49, *p* = 0.03). During follow-up (T2), the CD group exhibited the following significant correlations: TIMP-1 and CRP (R = 0.51, *p* = 0.03), TIMP-1 and fCal (R = 0.61, *p* = 0.007) (Supplementary Table S2a,b).

### 2.4. Changes in MMP-9, TIMP-1 and MMP-9/TIMP-1 Ratio According to Clinical Response at 6 Months in Patients with IBD

In the next phase of the study, patients were divided into groups based on PUCAI/PCDAI scores at T2 (Table 3). Clinical remission was achieved by approximately 60% of patients after six months. In the group of patients who achieved clinical remission after 6 months, there were 24 children with UC and 14 with CD. In those who achieved clinical remission, a significant decrease in MMP-9 and TIMP-1 levels was observed. In contrast, no significant changes in MMP-9 or TIMP-1 were noted in patients who did not achieve remission. At T2, no significant differences in MMP-9, TIMP-1, or the MMP-9/TIMP-1 ratio were observed between the two groups. Analysis of changes between T2 and T0 (ΔT2 − T0) revealed that ΔMMP-9 did not differ significantly between patients with and without clinical remission (−35.74% [−87.63% to 153.30%] vs. −23.91% [−81.80% to 193.16%]; *p* = 0.50), whereas ΔTIMP-1 was significantly greater in patients who achieved remission (−22.00% [−57.25% to 19.419%] vs. −11.44% [−44.94% to 131.17%]; *p* = 0.02). ROC analysis demonstrated that ΔTIMP-1 discriminated between patients who achieved clinical remission and those who did not, with an AUC of 0.682 (95% CI: 0.54–0.823; *p* = 0.01) and an optimal cut-off value of –13.42%. For changes between T1 and T0 (ΔT1 − T0), no significant differences were observed for MMP-9 (−36.14% [−84.31% to 239.35%] vs. −1.03% [−62.81% to 194.77%]; *p* = 0.25) or TIMP-1 (−15.40% [−55.33% to 176.96%] vs. −1.01% [−38.46% to 146.43%] *p* = 0.07).

As expected, CRP improved in both groups. When comparing both groups at T0, no significant difference was observed. It is important to note that the patients were analyzed independently of the treatment regimen used, which may have significant implications for the interpretation of the results and requires caution when drawing conclusions. With regard to treatment, no differences in CRP levels were observed between the group that achieved clinical remission and the group that did not. However, in the remission group, there was a significant increase in the use of 5-aminosalicylic acid (5-ASA).

### 2.5. Changes in MMP-9, TIMP-1, and the MMP-9/TIMP-1 Ratio According to fCal Levels at 6 Months in Patients with IBD

As mentioned earlier, fCal-defined biochemical remission, defined as an fCal level <250 µg/g, was achieved at time point T2 in almost half of the patients (19 children with ulcerative colitis and 10 with Crohn’s disease) (Table 4). Baseline concentrations of MMP-9, TIMP-1, and the MMP-9/TIMP-1 ratio did not differ significantly between patients who achieved and those who did not achieve fCal-defined biochemical remission. In both the high-fCal and low-fCal groups, MMP-9 and TIMP-1 levels decreased over time, whereas the MMP-9/TIMP-1 ratio did not show a significant reduction in the high-fCal group. Additionally, among standard inflammatory and clinical markers, a trend toward normalization was observed for PUCAI and CRP. Detailed information is presented in Table 4.

Overall, changes between T2 and T0 (ΔT2 − T0) in MMP-9 (*p* = 0.28) and TIMP-1 (*p* = 0.37) did not differ significantly between patients with and without fCal-defined biochemical remission. Similarly, changes between T1 and T0 (ΔT1 − T0) showed no significant differences between the groups for MMP-9 (*p* = 0.54) or TIMP-1 (*p* = 0.77). These findings indicate that the changes in MMP-9 and TIMP-1 concentrations over time did not differ significantly according to fCal-defined biochemical remission status.

### 2.6. Changes in MMP-9, TIMP-1, and the MMP-9/TIMP-1 Ratio According to CRP Levels at Baseline in Patients with IBD

The next stage of the study was to evaluate changes in MMP-9 and TIMP-1 concentrations depending on baseline CRP levels. Patients were stratified according to baseline CRP levels into two groups: CRP < 5 mg/L (n = 29; CD: 4, UC: 25) and CRP > 5 mg/L (n = 30; UC: 15, CD: 15) (Table 5). The cut-off value of 5 mg/L was chosen as it represents the upper limit of normal in our laboratory and is widely used to differentiate between normal and elevated CRP levels in clinical practice. Baseline concentrations of CRP were used for the analysis, as after 6 months only seven patients in the entire study cohort had elevated CRP levels. Such a large disproportion between groups made statistical analysis infeasible. At baseline (T0), the two groups differed in TIMP-1 concentrations, with higher values observed in the group with elevated CRP. Other parameters differentiating the groups at T0 included ESR, ferritin, albumin and fCal. After 6 months of follow-up, comparable values of MMP-9, TIMP-1 and the MMP-9/TIMP-1 ratio were observed between the groups. Only CRP remained significantly higher in the group with baseline CRP > 5 mg/L, although the median values in both groups did not exceed the upper limit of normal. In the group with low baseline CRP, significant changes over the 6-month observation period were noted in MMP-9, MMP-9/TIMP-1 ratio, PUCAI/PCDAI scores, CRP and fCal. In the group with baseline CRP > 5 mg/L, significant changes were observed in MMP-9, TIMP-1, CRP and fCal.

### 2.7. Changes in MMP-9, TIMP-1, and the MMP-9/TIMP-1 Ratio According to Baseline Disease Status

**Figure 1:**
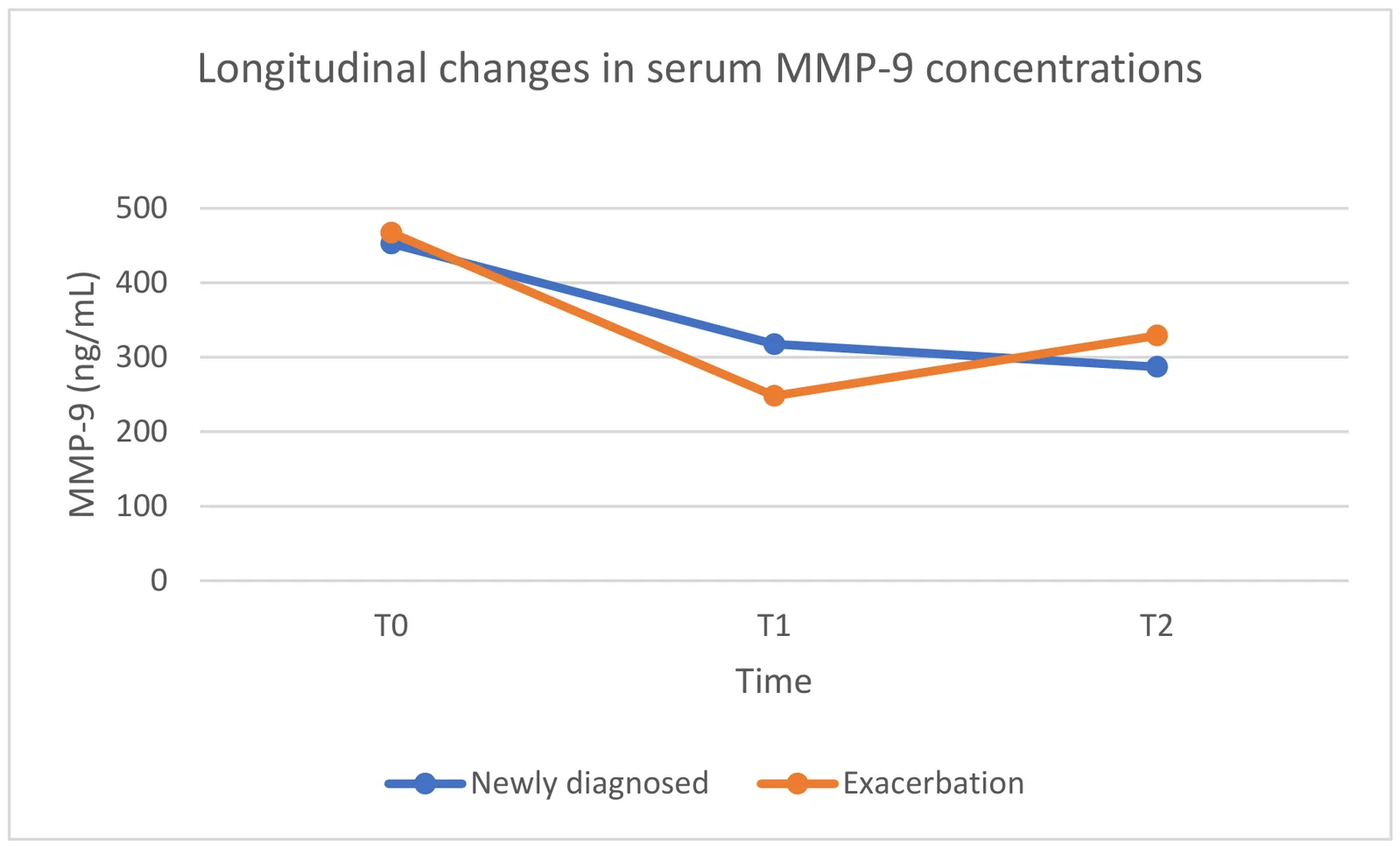
Longitudinal changes in serum MMP-9 concentrations during the six-month follow-up in children with inflammatory bowel disease. Data are presented as medians for the entire IBD cohort and separately for treatment-naïve patients (newly diagnosed) and patients with disease exacerbation at T0, T1, and T2.

**Figure 2:**
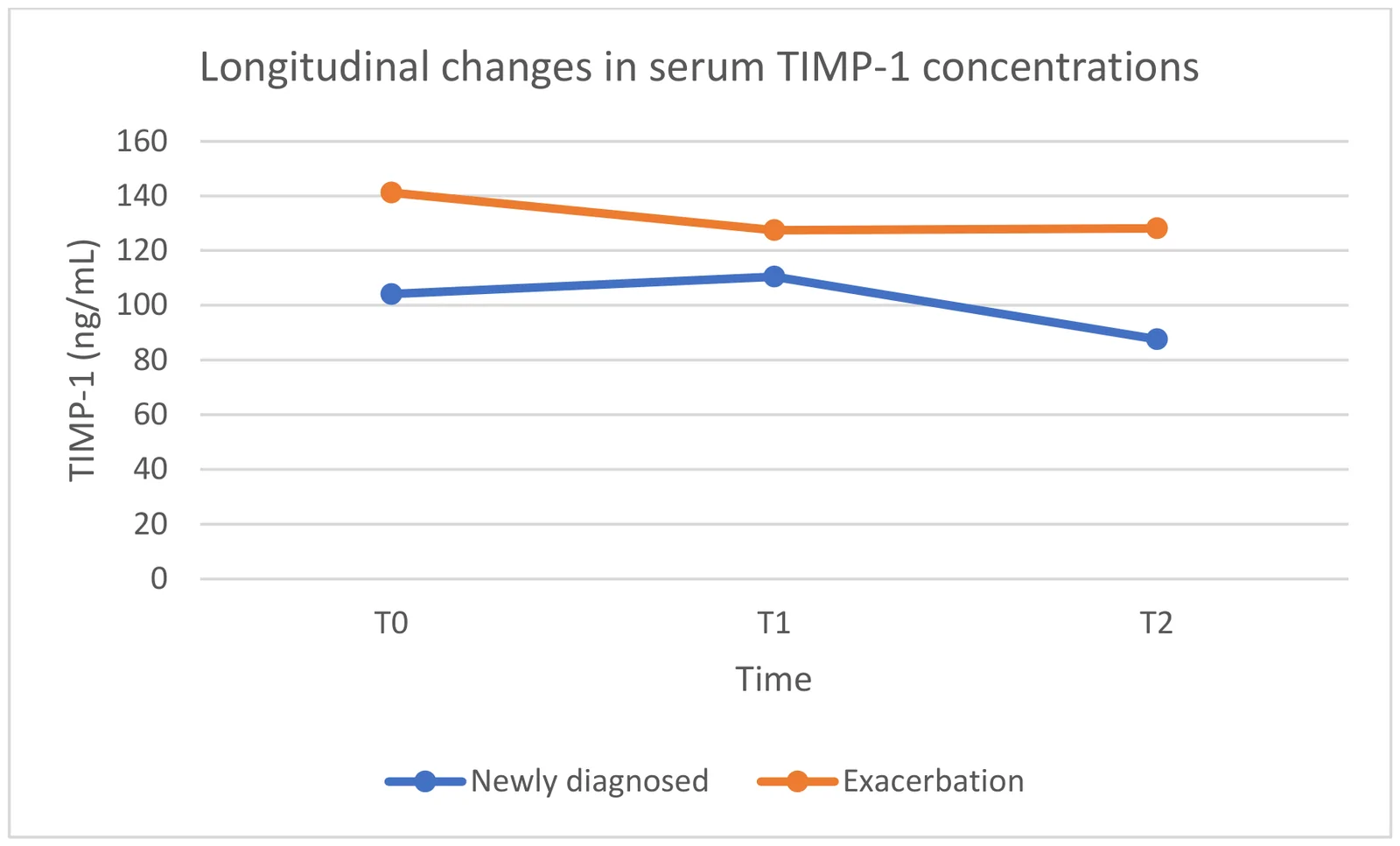
Longitudinal changes in serum TIMP-1 concentrations during the six-month follow-up in children with inflammatory bowel disease. Data are presented as medians for the entire IBD cohort and separately for treatment-naïve patients (newly diagnosed) and patients with disease exacerbation at T0, T1, and T2.

In the IBD group, two-thirds of the patients were newly diagnosed and had not received any prior treatment (Table 6). In treatment-naïve patients, a significant decrease in MMP-9 and TIMP-1 levels was observed during the follow-up period. In previously treated patients, apart from the decrease in MMP-9 and TIMP-1, significant changes were also noted in the MMP-9/TIMP-1 ratio. Regarding standard inflammatory markers, both groups demonstrated a trend toward normalization of parameters such as CRP and fCal, as well as clinical activity indices (PUCAI/PCDAI). At baseline (T0), no significant differences in MMP-9 and TIMP-1 concentrations were found between the two groups. In both treatment-naïve and exacerbation groups, MMP-9 and TIMP-1 concentrations changed significantly over the six-month follow-up; however, no significant differences between the groups were observed at baseline (T0) or after six months (T2) (Table 6). The longitudinal changes in MMP-9 and TIMP-1 concentrations in treatment-naïve patients and patients with disease exacerbation are presented in Figure 1 and Figure 2, respectively.

## 3. Discussion

In the present study, we demonstrated that the protease–inhibitor axis is significantly activated in pediatric patients with active IBD, including both newly diagnosed cases and those experiencing disease flare. In the previous studies we demonstrated the potential utility of MMPs in newly diagnosed IBD pediatric patients. In the study by Daniluk et al., children with newly diagnosed CD showed significantly higher levels of MMP-9 and TIMP-1 compared with controls, both in serum and fecal samples. Moreover, both MMP-9 and TIMP-1 were useful in differentiating patients according to disease characteristics, including disease location and MRI findings [14]. Similarly, among children with newly diagnosed UC, significantly higher serum and fecal concentrations of MMP-9 and TIMP-1 were observed compared to the control group. Importantly, children with more extensive disease presented with higher levels of these markers [15]. In our study, at baseline, circulating concentrations of MMP-9 and TIMP-1 in IBD patients were significantly elevated compared with controls, while the MMP-9/TIMP-1 ratio remained unchanged, indicating a proportional increase in both components of the protease–inhibitor axis. As previous research has focused primarily on a single time point at diagnosis, our study can be considered a continuation and extension of these observations.

To the best of our knowledge, no studies have applied a design comparable to ours, particularly with respect to assessing these biomarkers across different phases of active disease. In our study, both MMP-9 and TIMP-1 levels decreased significantly over the six-month follow-up. These findings suggest that these markers may reflect dynamic changes in inflammatory activity rather than baseline disease status. The magnitude of the change in MMP-9 did not differ significantly according to clinical remission status, whereas a significant difference in TIMP-1 percentage change was observed between patients who achieved clinical remission and those who did not. This finding should be considered exploratory and interpreted with caution, particularly given the multiple subgroup comparisons and potential baseline differences between the groups.

Moreover, after stratification of patients at baseline according to CRP levels, higher TIMP-1 concentrations were observed in patients with elevated CRP. This may reflect a response to increased inflammatory activity, and TIMP-1 is known to be upregulated by pro-inflammatory cytokines such as IL-6, which also drive CRP production [16]. Furthermore, the decrease in TIMP-1 levels observed during follow-up in the high-CRP group may be associated with a reduction in inflammatory activity. MMP-9 has been investigated in various inflammatory diseases in both pediatric and adult populations. Takeshita et al. analyzed changes in MMP-9 concentrations in children with Kawasaki disease across different stages of the disease, from the acute phase to the convalescent phase. Measurements were performed from the onset of the disease until day 35 of follow-up. The authors reported the highest MMP-9 levels during the acute phase, lower levels during the subacute phase and the lowest levels during the convalescent phase [17]. In our study, elevated MMP-9 levels were also observed during the acute phase of the inflammatory disease, with a decreasing trend over the course of the disease. These findings support the concept that MMP-9 may reflect the activity of the inflammatory process. Changes in the concentrations of both MMP-9 and TIMP-1 were also monitored in a study conducted in adult patients with rheumatoid arthritis [18]. In that study, the changes were evaluated in relation to treatment with infliximab, a therapy that is also widely used in the treatment of IBD. During treatment, the concentrations of both MMP-9 and TIMP-1 decreased significantly. Two observations from that study are particularly interesting. Although MMP-9 levels gradually decreased throughout the 52-week follow-up period, the most pronounced decline was observed after the second anti-tumor necrosis factor-α (anti-TNF-α) infusion. In contrast, despite an initial decrease in TIMP-1 levels, a tendency toward an increase back toward baseline values was observed in the later weeks of the study. In our study, we did not observe a similar increase in TIMP-1 levels in patients with IBD. However, extending the duration of follow-up in our study might have provided a more comprehensive insight into the dynamics of the protease–inhibitor balance.

In the limited number of studies investigating longitudinal changes in MMP-9 and TIMP-1 in IBD, the analyses were usually focused on the effect of specific therapies on the concentrations of these biomarkers. In contrast, the primary aim of our study was to evaluate the changes in MMP-9 and TIMP-1 levels during a six-month follow-up period independent of a specific treatment regimen, reflecting real-world clinical practice in which patients were treated according to current clinical guidelines. In a study conducted in a pediatric IBD population, the effects of steroids and anti-TNF-α treatment on the concentrations of several MMPs, including MMP-9 and their inhibitors were evaluated [19]. Measurements were performed after 4 weeks of steroids therapy and after 2 weeks of anti-TNF-α treatment. No statistically significant changes in serum MMP-9 levels were observed during both therapies. In contrast, TIMP-1 levels decreased significantly during usage of steroids, and in patients receiving anti-TNF-α agents the change did not reach statistical significance. This study demonstrates that the type of treatment may be an important factor influencing MMP-9 and TIMP-1 concentrations. However, it should be noted that the follow-up period in this study was relatively short. In studies conducted in adult patients with CD, the effect of infliximab therapy on both serum MMP-9 concentrations and intestinal MMP-9 expression assessed by immunohistochemistry has been investigated [20]. A general trend toward decreased serum MMP-9 levels was observed in infliximab-treated patients. Patients who responded to treatment tended to have slightly lower MMP-9 levels between weeks 6 and 18 compared with placebo-treated patients and those who did not respond to therapy, although the differences did not reach statistical significance. Immunohistochemical analyses of follow-up biopsies further demonstrated a reduction in the intensity of MMP-9 staining as early as day 3 after treatment, with an even more pronounced decrease observed after two weeks of therapy. However, these immunohistochemical findings should be interpreted with caution, as the study was conducted in a relatively small cohort of patients (fewer than 10 individuals) [20]. Studies involving larger patient cohorts, with particular consideration of the type of treatment administered and with longer observation periods, would help provide a more comprehensive understanding of MMP-9 and TIMP-1 dynamics in IBD.

Importantly, direct comparisons between the groups showed no significant differences in the percentage changes in MMP-9 (*p* = 0.28) or TIMP-1 (*p* = 0.37). These findings therefore do not demonstrate that the magnitude of MMP-9 or TIMP-1 reduction is associated with fCal-defined biochemical remission. Importantly, fCal was used as a biochemical marker of remission in our study, and follow-up endoscopic assessment of mucosal healing was not performed. It would also be interesting to assess MMP-9 and TIMP-1 levels directly in intestinal tissue, as our study was based only on serum measurements, which may not fully reflect local processes in the bowel mucosa.

MMP-9 and TIMP-1 have also been investigated as potential biomarkers associated with mucosal healing in IBD. In a study evaluating the complex neutrophil gelati-nase–associated lipocalin (NGAL)–MMP-9 as a surrogate marker of mucosal healing in patients with CD, significantly higher baseline levels of NGAL–MMP-9 were observed in patients with active disease compared with healthy controls. Following treatment, NGAL–MMP-9 levels decreased significantly in patients who achieved complete mucosal healing, whereas a less pronounced reduction was observed in patients without mucosal healing [21]. In our study, patients who achieved fCal-defined biochemical remission showed significant reductions in MMP-9, TIMP-1 and the MMP-9/TIMP-1 ratio over the study period. However, MMP-9 and TIMP-1 levels also decreased significantly in patients who did not achieve fCal-defined biochemical remission. Importantly, direct comparisons between the groups showed no significant differences in the percentage changes in MMP-9 or TIMP-1. These findings therefore do not demonstrate that the magnitude of MMP-9 or TIMP-1 reduction is associated with fCal-defined biochemical remission. It would also be interesting to assess MMP-9 and TIMP-1 levels directly in intestinal tissue, as our study was based only on serum measurements, which may not fully reflect local processes in the bowel mucosa. In another study, TIMP-2, a metalloproteinase inhibitor that was not assessed in our study, was suggested as a potential prognostic marker, as the percentage reduction in serum TIMP-2 levels was associated with both early (14-week) and long-term remission [22]. In our cohort of IBD patients, we observed that the magnitude of TIMP-1 reduction differed significantly between patients who achieved clinical remission and those who did not, with the change calculated as the difference between baseline and the 6-month follow-up. Further studies are needed to determine whether MMP-9 and TIMP-1 may indeed serve as useful biomarkers for predicting disease course and supporting therapeutic decision in patients with IBD.

The main strength of our study is the novelty of evaluating MMP-9 and TIMP-1 at multiple time points in children with IBD and subsequently relating these parameters to clinical disease activity and fCal-defined biochemical remission. This longitudinal approach allowed us to assess the dynamics of these biomarkers during follow-up. However, several limitations should also be acknowledged. The relatively small sample size limited the possibility of performing additional subgroup analyses, such as evaluating changes in biomarker levels separately in patients with CD and UC. Similarly, we were unable to perform analyses according to the type of treatment administered. We are aware that our findings should be confirmed in larger studies with appropriate sample size calculation and statistical power. Another limitation is the lack of comparison with children affected by other intestinal diseases, which could further strengthen the clinical relevance of the observed result. An important consideration is that the control group consisted of children with functional abdominal pain and normal fCal rather than healthy children. Recruitment of truly healthy pediatric controls in a hospital-based setting is challenging and may be difficult to perform ethically and practically. Therefore, the observed differences in MMP-9 and TIMP-1 concentrations should be interpreted as differences between children with IBD and children without evidence of intestinal inflammation, rather than differences compared with a healthy pediatric population.

## 4. Materials and Methods

### 4.1. Patients and Study Design

This prospective study included children with newly diagnosed or relapsing IBD (UC and CD). The control group consisted of children with functional abdominal pain and normal fCal levels. Participants were enrolled between 2020 and 2022. IBD diagnosis was established according to European Society for Pediatric Gastroenterology, Hepatology and Nutrition criteria, based on clinical, endoscopic, histological, and radiological findings [1]. Detailed methodological procedures have been described previously [7,23] and are summarized here. For the purposes of the study, three assessment time points were selected: at the time of diagnosis (T0), 3 months (T1) and 6 months (T2) after diagnosis and remission-inducing treatment. Disease activity was assessed using validated indices (PCDAI for CD and PUCAI for UC) according to established criteria [24,25]. Disease phenotype was classified using the Paris classification. Endoscopic severity was evaluated using standard scoring systems (Mayo Endoscopic Score for UC and Simple Endoscopic Scale for Crohn’s Disease (SES-CD) for CD) [26,27]. Once the diagnosis was made, the patients were treated according to current recommendations for that time [28,29,30,31]. Anti-TNF-α treatment was administered according to the Summary of Product Characteristics of the respective medicinal products. IFX was administered intravenously at a dose of 5 mg/kg at weeks 0, 2, and 6, followed by maintenance administration every 8 weeks. ADA was administered subcutaneously to patients weighing >40 kg at a dose of 80 mg at week 0, followed by 40 mg at week 2 and 40 mg every 2 weeks as maintenance therapy. Patients with UC received infliximab only, whereas patients with CD received either IFX or ADA according to their clinical situation. Additionally, after 6 months of treatment (T2), children with UC and CD were stratified into subgroups according to clinical activity indices (PUCAI for UC and PCDAI for CD; remission < 10 points vs. active disease ≥ 10 points) and fecal calprotectin levels (fCal-defined biochemical remission < 250 µg/g vs. no fCal-defined biochemical remission ≥ 250 µg/g) [32,33]. All patients were reassessed at T2, six months after the initial evaluation. This grouping allowed for a descriptive comparison of serum MMP-9 and TIMP-1 concentrations in relation to clinical and biochemical disease activity. Furthermore, patients were analyzed according to baseline CRP levels, as well as disease status at inclusion (newly diagnosed patients vs. those experiencing disease exacerbation). The study was initially planned to include 60 children with IBD. One patient did not attend the scheduled visit and therefore did not have a blood sample collected for the study.

### 4.2. Samples Collection and Measurements of Routine Blood Tests, MMP-9, TIMP-1 and fCal

All participants underwent a physical examination. All included patients had usable serum samples available at all three study time points (T0, T1 and T2). CRP was measured in the hospital laboratory on the day of blood sample collection. Blood samples for assessment of MMP-9 and TIMP-1 were collected into serum tubes without anticoagulant and allowed to clot for approximately 2 h at room temperature. The samples were then centrifuged at 1000× *g* for 20 min. The obtained serum was aliquoted and stored at −80 °C until further analysis. The storage period ranged from 6 to 112 weeks. Samples underwent one freeze–thaw cycle before analysis. Serum concentrations of MMP-9 and TIMP-1 were determined using double-antibody sandwich enzyme-linked immunosorbent assay (ELISA) kits according to the manufacturer’s instructions (Cloud-Clone Corp., Katy, TX, USA; MMP-9, SEA553Hu; TIMP-1, SEA552Hu). The ratio of serum MMP-9 to TIMP-1 concentrations was calculated after determining the respective serum concentrations.

For interpretation of routine laboratory parameters, the reference range for C-reactive protein (CRP) was <5 mg/L, while a fecal calprotectin (fCal) concentration <250 µg/g was considered indicative of biochemical remission. No established pediatric reference ranges are available for serum MMP-9 and TIMP-1 concentrations; therefore, a control group was included to provide a comparative reference population for these biomarkers.

### 4.3. Statistical Analysis

Data analysis was performed using the Statistica software version 13.3 (TIBCO Software Inc., Palo Alto, CA, USA). Continuous variables are reported as medians (range) and compared using the Mann–Whitney U test, while categorical variables are presented as numbers and percentages and analyzed with the Chi-square test. Between-group comparisons were performed at the same time points. Within-group changes across T0, T1 and T2 were assessed using the Friedman test for continuous variables and Cochran’s Q test with Chi-square test for categorical variables. Bonferroni correction was applied for post hoc pairwise comparisons. Spearman’s rank correlation was applied to evaluate associations between variables. The diagnostic performance of MMP-9 and TIMP-1 was assessed using receiver operating characteristic (ROC) curve analysis. To calculate the sensitivity, the specificity and the accuracy of MMP-9 and TIMP-1, the cut-off values were selected based on the Youden index maximization criterion. A statistical significance was noticed with *p* value < 0.05.

### 4.4. Ethical Consideration

Written informed consent was obtained from the parents of all patients and by patient in the case of a child aged ≥16 years. The study was approved by the Bioethics Committee of the Medical University of Bialystok (approval numbers APK.002.409.2020, date: 17 December 2020 and APK.002.252.2025, date: 26 June 2025) prior to patient recruitment, and the study is in accordance with the Declaration of Helsinki.

## 5. Conclusions

In conclusion, we demonstrated that serum concentrations of MMP-9 and TIMP-1 change over a six-month period in patients with IBD. Moreover, these markers reflect inflammatory activity and do not appear to depend on disease duration. Although an overall decreasing trend in MMP-9 and TIMP-1 concentrations was observed over time, direct between-group comparisons did not demonstrate significant differences in the magnitude of MMP-9 or TIMP-1 changes according to fCal-defined biochemical remission. A significant difference in TIMP-1 percentage change was observed according to clinical remission status; however, this finding should be considered exploratory and interpreted with caution.

## Figures and Tables

**Table 1:** Clinical and demographic characteristics of children with UC and CD at the time of inclusion in the study (T0).

Parameters	CD (n = 19, 32.20%)	UC (n = 40, 67.80%)
Age years median (range)	15 (7–17)	14 (7–17)
Male n (%)	9 (47.37%)	18 (45.0%)
Disease activity index median (range)	PCDAI: 37.5 (10–72.5)	PUCAI: 50 (10–80)
IBD activity:		
Remission n (%)	0 (0.0%)	0 (0.0%)
Mild n (%)	6 (31.58%)	13 (32.5%)
Moderate n (%)	5 (26.31%)	17 (42.5%)
Severe n (%)	8 (42.11%)	10 (25.0%)
Location—L1/L2/L3 n (%)	4 (21.05%)/5 (26.32%)/10 (52.63%)	NA
Behavior—B1/B2 n (%)	10 (52.63%)/9 (47.37%)	NA
Growth—G1/G0 n (%)	5 (26.32%)/14 (73.68%)	NA
Extent—E1/E2/E3/E4 n (%)	NA	7 (17.5%)/10 (25.0%)/9 (22.5%)/14 (35.0%)
SES-CD, median (range)	13 (6–24)	NA
Mayo endoscopic score I/II/III n (%)	NA	6 (15.0%)/16 (40.0%)/18 (45.0%)
Newly diagnosed patients n (%)	12 (63.16%)	27 (67.5%)
Exacerbations n (%)	7 (36.84%)	13 (32.5%)
Treatment:		
Naïve n (%)	12 (63.16%)	27 (67.5%)
Nutritional therapy n (%)	2 (10.53%)	NA
Nutritional therapy + AZA/6-MP n (%)	1 (5.26%)	NA
AZA/6-MP n (%)	4 (21.05%)	0 (0.0%)
5-ASA n (%)	NA	9 (22.5%)
5-ASA + AZA/6-MP n (%)	NA	4 (10.0%)

UC—ulcerative colitis, CD—Crohn’s disease, NA: not applicable, L1—distal 1/3 ileum, L2—colonic, L3—ileocolonic, B1—non-stricturing, non-penetrating, B2—stricturing, G0—no evidence of growth delay, G1—growth delay, E1—ulcerative proctitis, E2—left-sided colitis, E3—extensive colitis (hepatic flexure distally), E4—pancolitis, SES-CD—simple endoscopic score for Crohn’s disease, AZA—azathioprine, 6-MP—6-mercaptopurine, 5-ASA—5-aminosalicylic acid, NA—not applicable.

**Table 2:** MMP-9, TIMP-1, the MMP-9/TIMP-1 ratio and other biochemical and clinical parameters were assessed at T0, T1 and T2 in the IBD group, UC group, CD group and control group (Ctr). All data are presented as medians with minimum and maximum values.

**Parameters**	**IBD at T0 (n = 59)**	**IBD at T1 (n = 59)**	**IBD at T2 (n = 59)**	**Ctr (n = 27)**	**T0 vs. T1 vs. T2**
MMP-9 (ng/mL)	459.2 (145.9–1499.7) **	314.3 (73.7–1696.4)	309.9 (73.4–1100.5)	273.1 (136.6–456.3)	<0.001 ##
TIMP-1 (ng/mL)	127.80 (42.083–260.30) **	111.00 (39.015–276.50) *	95.600 (44.494–183.10)	88.307 (42.429–16.205)	<0.001 #, ##
MMP-9/TIMP-1 ratio	3.3 (0.9–16.2)	3.2 (0.8–16.8)	2.86 (0.9–16.3)	2.9 (1.2–6.8)	0.006 ##
CRP (mg/L)	5.08 (0.6–119.35) **	0.6 (0.6–63.88) **	0.65 (0.6–60.0) **	0.6 (0.3–4.6)	<0.001 #, ##
CRP < 5 md/L n (%)	29 (49.15%)	48 (81.36%)	52 (88.14%)	27 (100.00%)	<0.001 #, ##
fCal (µg/g)	2551 (20–4730) **	463 (20–4760) **	337 (18–3805) **	29 (16–99)	<0.001 #, ##
fCal < 250 µg/g n (%)	3 (5.08%)	26 (44.07%)	29 (49.15%)	27 (100.00%)	<0.001 #, ##
PCDAI/PUCAI	45 (10–80)	10 (0–65)	5 (0–65)	NA	<0.001 #, ##, ###
PCDAI/PUCAI < 10 n (%)	0 (0.0%)	27 (45.76%)	38 (64.41%)	NA	NA
**Parameters**	**UC at T0 (n = 40)**	**UC at T1 (n = 40)**	**UC at T2 (n = 40)**	**Ctr (n = 27)**	**T0 vs. T1 vs. T2**
MMP-9 (ng/mL)	505.2 (175.1–1499.7) **	321.25 (73.7–1696.4)	315.70 (88.70–1100.50)	273.1 (136.6–456.3)	0.04 ##
TIMP-1 (ng/mL)	100.742 (42.083–263.00)	108.703 (39.015–224.90)	86.872 (44.494–183.11)	88.307 (42.429–162.05)	0.01 ##
MMP-9/TIMP-1 ratio	3.6 (1.3–16.2)	3.25 (1.0–16.8)	3.0 (1.0–16.3)	2.9 (1.2–6.8)	0.70
CRP (mg/L)	2.43 (0.6–119.35) **	0.6 (0.6–21.25)	0.61 (0.60–60.0) *	0.6 (0.3–4.6)	<0.001 #, ##
CRP < 5 md/L n (%)	25 (62.50%)	35 (87.50%)	36 (90.00%)	27 (100.0%)	0.004 #, ##
fCal (µg/g)	2510 (100–3975) **	463 (20–4760) **	397 (18–3805) **	29 (16–99)	0.001 #, ##
fCal < 250 µg/g n (%)	2 (5.00%)	17 (42.50%)	19 (47.50%)	27 (100.0%)	<0.001#, ##
PUCAI	50 (10–80)	5 (0–65)	5 (0–65)	NA	<0.001 #, ##
PUCAI < 10 n (%)	0 (0.0%)	21 (52.50%)	24 (60.00%)	NA	NA
**Parameters**	**CD at T0 (n = 19)**	**CD at T1 (n = 19)**	**CD at T2 (n = 19)**	**Ctr (n = 27)**	**T0 vs. T1 vs. T2**
MMP-9 (ng/mL)	392.0 (145.9–1388.7) *	290.3 (101.7–665.4)	270.8 (73.4–655.8)	273.1 (136.6–456.3)	0.002 ##
TIMP-1 (ng/mL)	131.20 (78.937–229.20) **	116.014 (64.062–276.50) **	113.60 (58.873–171.70) *	88.307 (42.429–162.05)	0.002 #, ##
MMP-9/TIMP-1 ratio	2.4 (0.9–10.6)	2.4 (0.8–5.8)	1.9 (0.9–05.7) **	2.9 (1.2–6.8)	0.15
CRP (mg/L)	24.25 (0.6–67.11) **	1.22 (0.66–63.88) **	1.02 (0.6–7.02) *	0.6 (0.3–4.6)	<0.001 #, ##
CRP < 5 mg/L n (%)	4 (21.05%)	13 (68.42%)	16 (84.21%)	27 (100.00%)	<0.001 #, ##
fCal (µg/g)	2696 (20–4730) **	350 (20–3401) **	288 (20–2597) **	29 (16–99)	<0.001 #, ##
fCal < 250 µg/g n (%)	1 (5.26%)	9 (47.37%)	10 (52.63%)	27 (100.00%)	0.004 #, ##
PCDAI	37.5 (10–72.5)	15 (0–55)	5 (0–55)	NA	<0.001 #, ##, ###
PCDAI < 10 n (%)	0 (0.00%)	6 (31.58%)	14 (73.68%)	NA	NA

* *p* < 0.05; vs. control group, ** *p* < 0.01; vs. control group, # T0 vs. T1, ## T0 vs. T2, ### T1 vs. T2. MMP-9—metalloproteinase-9, TIMP-1—tissue inhibitor of metalloproteinase-1, CRP—C-reactive protein, fCal—fecal calprotectin, PUCAI—Pediatric activity index, PCDAI—pediatric Crohn’s disease activity index, NA—not applicable.

**Table 3:** Changes in marker levels according to clinical disease activity assessed by PUCAI/PCDAI levels at T2. All values are presented as medians with minimum and maximum values. Comparisons within the same groups at T0, T1 and T2 were assessed using the Friedman test with post hoc analysis or Cochran’s Q test with Chi-square test. Comparisons between groups at T0 and T2 were assessed using the Mann–Whitney U test.

	PUCAI/PCDAI < 10 at T2 (n = 38; 64.41%)				PUCAI/PCDAI ≥ 10 at T2 (n = 21; 35.59%)					
Parameters	IBD T0	IBD T1	IBD T2	*p* T0 vs. T1 vs. T2	IBD T0	IBD T1	IBD T2	*p* T0 vs. T1 vs. T2	*p* T0 (<10) vs. T0 (≥10)	*p* T (<10) vs. T0 (≥10)
Naive n (%)	27 (71.05%)	0 (0.0%)	0 (0.0%)	NA	12 (57.14%)	0 (0.0%)	0 (0.0%)	NA	0.28	NA
Nutritional therapy n (%)	1 (2.63%)	2 (5.26%)	0 (0.0%)	NA	1 (4.76%)	1 (4.76%)	0 (0.0%)	NA	0.67	NA
5-ASA n (%)	4 (10.53%)	16 (42.11%)	14 (36.84%)	<0.001 #, ##	5 (23.81%)	8 (39.0%)	6 (28.57%)	0.53	0.17	0.52
5-ASA/Nutritional therapy +IMs +/−steroids n (%)	6 (15.79%)	11 (28.95%)	8 (21.05%)	0.49	3 (14.28%)	7 (33.33%)	6 (28.57%)	0.37	0.88	0.51
Anti-TNF-α n (%)	0 (0.0%)	9 (23.68%)	16 (42.11%)	NA	0 (0.0%)	5 (23.81%)	9 (42.86%)	NA	NA	0.32
PUCAI/PCDAI	37.5 (10–75)	5 (0–65)	0 (0–7.5)	<0.001 #, ##, ###	50 (10–80)	15 (0–55)	15 (10–65)	<0.001 #,##	0.30	<0.001
PCDAI/PUCAI < 10 n (%)	0 (0.00%)	20 (52.63%)	38 (100.00%)	NA	0 (0.00%)	7 (33.33%)	0 (0.00%)	NA	NA	NA
MMP-9 (ng/mL)	478.7 (178.7 –1499.7)	315.15 (73.7–1696.4)	307.95 (85.6–1100.5)	0.001 ##	350.3 (145.9–841.3)	314.3 (120.7–1144.7)	315.7 (73.4–665.2)	0.16	0.15	0.63
TIMP-1 (ng/mL)	131.25 (50.012–260.30)	113.201 (39.015–276.50)	93.3775 (44.838–183.10)	<0.001 #, ##	93.967 (42.083–208.80)	110.50 (48.662–224.90)	90.90 (44.494–183.10)	0.25	0.09	0.66
MMP-9/TIMP-1 ratio	3.5 (1.1–16.2)	3.3 (0.8–16.8)	2.7 (1.0–16.3)	0.08	3.2 (0.9–11.2)	3.2 (0.8–9.1)	2.9 (0.9–9.5)	0.06	0.88	0.75
CRP (mg/L)	5.79 (0.60–63.95)	0.67 (0.6–63.88)	0.78 (0.6–10.59)	<0.001 #, ##	1.81 (0.6–119.35)	0.6 (0.6–41.41)	0.6 (0.6–60.0)	0.001 #, ##	0.25	0.28
CRP < 5 md/L n (%)	16 (42.11%)	30 (78.95%)	34 (89.47%)	<0.001 #, ##	13 (61.90%)	18 (85.71%)	18 (85.71%)	0.04	0.15	0.67
fCal (µg/g)	2509 (20–3975)	239 (20–4760)	225 (20–3805)	<0.001 #, ##	2736 (100–4730)	1315 (20–3697)	868 (18–3568)	0.11	0.86	0.08
fCal < 250 µg/g n (%)	1 (2.63%)	20 (52.63%)	17 (44.74%)	<0.001 #, ##	2 (9.52%)	6 (28.57%)	12 (57.14%)	0.01 ##	0.25	0.36

# T0 vs. T1, ## T0 vs. T2, ### T1 vs. T2. MMP-9—metalloproteinase-9, TIMP-1—tissue inhibitor of metalloproteinase-1, CRP—C-reactive protein, fCal—fecal calprotectin, PUCAI—Pediatric activity index, PCDAI—pediatric Crohn’s disease activity index, 5-ASA—5-aminosalicylic acid, IMM—immunomudulator, anti-TNF-α—anti-tumor necrosis factor-α, NA—not applicable.

**Table 4:** Changes in marker levels according to achieved fCal-defined remission assessed by fCal levels at T2. All values are presented as medians with minimum and maximum values. Comparisons within the same groups at T0, T1 and T2 were assessed using the Friedman test with post hoc analysis or Cochran’s Q test with Chi-square test. Comparisons between groups at T0 and T2 were assessed using the Mann–Whitney U test.

	fCal <250 µg/g in T2 (n = 29; 49.15%)				fCal >250 µg/g in T2 (n = 30; 50.85%)					
Parameters	IBD T0	IBD T1	IBD T2	*p* T0 vs. T1 vs. T2	IBD T0	IBD T1	IBD T2	*p* T0 vs. T1 vs. T2	*p* T0 (fCal < 250 µg/g) vs. T0 (fCal > 250 µg/g)	*p* T2 (fCal < 250 µg/g) vs. T2 (fCal > 250 µg/g)
Naive n (%)	22 (75.86%)	0 (0.00%)	0 (0.00%)	NA	17 (56.67%)	0 (0.00%)	0 (0.00%)	NA	0.12	NA
Nutritional therapy n (%)	1 (3.45%)	3 (10.34%)	0 (0.00%)	NA	1 (3.33%)	0 (0.00%)	0 (0.00%)	NA	NA	NA
5-ASA n (%)	6 (20.69%)	12 (41.38%)	12 (41.38%)	0.08	3 (10.00%)	12 (40.00%)	8 (26.67%)	0.04 #	0.25	0.23
5-ASA/Nutritional therapy +IMs +/−steroids n (%)	0 (0.00%)	9 (31.03%)	4 (13.79%)	NA	9 (30.00%)	9 (30.00%)	10 (33.33%)	0.80	NA	0.08
Anti-TNF-α n (%)	0 (0.00%)	5 (17.24%)	13 (44.83%)	NA	0 (0.00%)	9 (30.00%)	12 (40.00%)	NA	NA	0.71
PUCAI/PCDAI	37.5 (10–80)	10 (0–65)	5 (0–20)	<0.001 #,##	50 (10–75)	10 (0–55)	5 (0–65)	<0.001 #, ##	0.41	0.07
PCDAI/PUCAI < 10 n (%)	0 (0.00%)	13 (44.83%)	21 (72.41%)	NA	0 (0.00%)	14 (46.67%)	17 (56.67%)	NA	NA	0.11
MMP-9 (ng/mL)	467.1 (145.9–1453.2)	314.15 (73.7–1696.4)	286.65 (73.4–1100.5)	0.005 ##	392.0 (175.1–1499.7)	314.3 (101.7–1144.7)	334.2 (88.7–935.1)	0.04 ##	0.48	0.22
TIMP-1 (ng/mL)	131.30 (64.143–260.30)	113.201 (62.70–224.90)	99.55 (46.154–183.10)	0.003 ##	112.599 (42.083–227.80)	111.00 (39.015–276.50)	90.90 (44.494–171.70)	0.01 ##	0.12	0.93
MMP-9/TIMP-1 ratio	3.2 (0.9–10.6)	3.2 (0.8–16.8)	2.2 (0.9–6.4)	0.01 ##	3.5 (1.1–16.2)	4.0 (0.8–15.5)	3.2 (1.0–16.3)	0.25	0.62	0.06
CRP (mg/L)	8.30 (0.6–63.95)	0.67 (0.6–23.4)	0.6 (0.6–10.59)	<0.001 #,##	3.24 (0.6–119.35)	0.6 (0.6–63.88)	0.77 (0.6–60.0)	<0.001 #, ##	0.32	0.22
CRP < 5 md/L n (%)	12 (41.38%)	23 (79.31%)	26 (89.66%)	<0.001 #, ##	17 (56.67%)	25 (83.33%)	26 (86.67%)	0.36	0.24	0.29
fCal (µg/g)	2568 (463–3975)	233 (20–2376)	44 (18–239)	<0.001 #,##	2532 (20–4730)	1273 (20–4760)	1401 (336–3805)	0.05	0.71	<0.001
fCal < 250 µg/g n (%)	1 (3.45%)	15 (51.72%)	29 (100.00%)	<0.001 #, ##, ###	2 (6.67%)	11 (36.67%)	0 (0.00%)	NA	0.52	NA

# T0 vs. T1, ## T0 vs. T2, ### T1 vs. T2. MMP-9—metalloproteinase-9, TIMP-1—tissue inhibitor of metalloproteinase-1, CRP—C-reactive protein, fCal—fecal calprotectin, PUCAI—Pediatric activity index, PCDAI—pediatric Crohn’s disease activity index, 5-ASA—5-aminosalicylic acid, IMM—immunomudulator, anti-TNF-α—anti-tumor necrosis factor-α, NA—not applicable.

**Table 5:** Changes in biomarker concentrations depending on CRP levels at T0 (<5 mg/L vs. >5 mg/L). All values are presented as medians with minimum and maximum values. Comparisons within the same groups at T0, T1, and T2 were assessed using the Friedman test with post hoc analysis or Cochran’s Q test with Chi-square test. Comparisons between groups at T0 and T2 were performed using the Mann–Whitney U test.

	CRP < 5 mg/L in T0 (n = 29; 49.15%)				CRP > 5 mg/L in T0 (n = 30; 50.85%)					
Parameters	IBD T0	IBD T1	IBD T2	*p* T0 vs. T1 vs. T2	IBD T0	IBD T1	IBD T2	*p* T0 vs. T1 vs. T2	*p* T0 (CRP < 5 mg/L) vs. T0 (CRP > 5 mg/L)	*p *T2 (CRP < 5 mg/L) vs. T0 (CRP > 5 mg/L)
Naive n (%)	16 (55.17%)	NA	NA	NA	23 (76.67%)	NA	NA	NA	0.08	NA
Nutritional therapy n (%)	1 (3.45%)	0 (0.00%)	0 (0.00%)	NA	1 (3.33%)	3 (10.0%)	0 (0.00%)	NA	0.57	NA
5-ASA n (%)	7 (24.14%)	14 (%)	10 (34.48%)	0.14	2 (6.67%)	10 (33.33%)	10 (33.33%)	0.004 #, ##	0.06	0.93
5-ASA/Nutritional therapy +IMs +/−steroids n (%)	5 (17.24%)	9 (31.03%)	10 (34.48%)	0.22	4 (13.33%)	9 (30.00%)	4 (13.33%)	0.47	0.68	0.93
Anti-TNF-α n (%)	0 (0.00%)	6 (20.69%)	9 (31.03%)	NA	0 (0.00%)	8 (26.67%)	16 (53.33%)	NA	NA	0.08
PUCAI/PCDAI	37.5 (10–80)	5 (0–50)	5 (0–65)	<0.001 #,##	52.5 (10–75)	15 (0–65)	5 (0–55)	<0.001 #, ##, ###	0.07	0.15
PCDAI/PUCAI < 10 n (%)	0 (0.00%)	17 (58.62%)	16 (55.17%)	NA	0 (0.00%)	10 (33.33%)	22 (73.33%)	NA	NA	0.15
MMP-9 (ng/mL)	428.9 (175.1–1419.8)	324.9 (73.7–1696.4)	315.7 (88.7–1100.5)	0.009 ##	459,9 (145.9–1499.7)	310.5 (130.4–1144.7)	296.1 (73.4–665.2)	0.02 ##	0.77	0.93
TIMP-1 (ng/mL)	96.177 (42.083–231.93)	100.742 (42.628 (276.50)	81.048 (44.494–183.10)	0.10	140.419 (50.012–260.30)	121.607 (39.015–224.90)	105.368 (48.149–183.10)	<0.001 #, ##	0.02	0.15
MMP-9/TIMP-1 ratio	3.8 (1.1–16.2)	3.1 (0.8–16.8)	2.9 (1.0–16.3)	0.01 ##	2.9 (0.9–11.8)	3.3 (1.0–15.5)	2.5 (0.9–7.6)	0.20	0.11	0.67
CRP (mg/L)	0.95 (0.6–4.04)	0.6 (0.6–63.88)	0.6 (0.6–7.19)	0.003 #	20.96 (5.08–119.35)	2.19 (0.6–41.41)	1.06 (0.6–60.0)	<0.001 #, ##	<0.001	0.03
CRP < 5 md/L n (%)	29 (100.00%)	28 (96.55%)	27 (93.10%)	0.37	0 (0.00%)	20 (66.67%)	25 (83.33%)	NA	NA	0.25
fCal (µg/g)	2394 (20–3975)	523 (20–4760)	456 (18–3805)	0.03 #, ##	2928 (1132–4730)	362 (920–3401)	225 (20–3461)	<0.001 #, ##	0.01	0.34
fCal < 250 µg/g n (%)	3 (20.34%)	13 (44.83%)	16 (55.17%)	0.001 #, ##	0 (0.00%)	13 (43.33%)	13 (43.33%)	NA	NA	0.36

# T0 vs. T1, ## T0 vs. T2, ### T1 vs. T2. MMP-9—metalloproteinase-9, TIMP-1—tissue inhibitor of metalloproteinase-1, CRP—C-reactive protein, fCal—fecal calprotectin, PUCAI—Pediatric activity index, PCDAI—pediatric Crohn’s disease activity index, 5-ASA—5-aminosalicylic acid, IMM—immunomodulator, anti-TNF-α—anti-tumor necrosis factor-α, NA—not applicable.

**Table 6:** Changes in biomarker concentrations in treatment-naïve patients versus patients with disease exacerbation. All values are presented as medians with minimum and maximum values. Comparisons within the same groups at T0, T1, and T2 were assessed using the Friedman test with post hoc analysis or Cochran’s Q test with Chi-square test. Comparisons between groups at T0 and T2 were performed using the Mann–Whitney U test.

	Treatment-Naïve at T0 (n = 39; 66.10%)				Disease Exacerbation at T0 (n = 20; 33.90%)					
Parameters	IBD T0	IBD T1	IBD T2	*p* T0 vs. T1 vs. T2	IBD T0	IBD T1	IBD T2	*p* T0 vs. T1 vs. T2	*p* T0 (Naive) vs. T0 (Exacerbation)	*p* T2 (Naive) vs. T0 (Exacerbation)
PUCAI/PCDAI	50 (10–75)	7.5 (0–65)	5 (0–65)	<0.001 #, ##	30 (10–80)	17.5 (0–55)	7.5 (0–20)	<0.001 ##	0.04	0.52
PCDAI/PUCAI < 10 n (%)	0 (0.00%)	20 (51.28%)	27 (69.23%)	NA	0 (0.00%)	7 (35.00%)	11 (55.00%)	NA	NA	0.28
MMP-9 (ng/mL)	452.6 (145.9–1499.7)	317.6 (73.7–1696.4)	287.1 (73.40–1100.5)	0.03 ##	467.1 (203.5–841.3)	248.3 (101.7–863.2)	329.2 (85.6–665.2)	0.005 ##	0.86	0.79
TIMP-1 (ng/mL)	104.103 (47.366–260.30)	110.50 (3015–229.0)	87.612 (44.494–183.10)	0.003 ##	141.20 (42.083–227.80)	127.50 (42.628–276.50)	128.15 (44.838–183.10)	0.01 ##	0.20	0.15
MMP-9/TIMP-1 ratio	3.5 (0.9–16.2)	3.5 (1.0–16.8)	2.9 (0.9–16.3)	0.10	2.9 (1.1–11.2)	2.4 (0.8–9.3)	2.6 (1.0–9.5)	0.03 ##	0.39	0.85
CRP (mg/L)	6.19 (0.6–119.35)	0.77 (0.60–34.73)	0.73 (0.6–60.0)	<0.001 #, ##	1.21 (0.60–67.11)	0.6 (0.6–63.88)	0.6 (0.6–6.26)	<0.001 #, ##	0.03	0.48
CRP < 5 md/L n (%)	16 (41.03%)	31 (79.49%)	34 (87.18%)	<0.001 #, ##	13 (65.00%)	17 (85.00%)	18 (90.00%)	0.05	0.08	0.75
fCal (µg/g)	2711 (100–4730)	427 (20–4760)	211 (20–3805)	<0.001 #, ##	1928 (20–3203)	600 (0–55)	472 (18–3555)	0.01 #, ##	0.02	0.53
fCal < 250 µg/g n (%)	1 (2.56%)	17 (43.59%)	17 (43.59%)	<0.001 #, ##	2 (10.00%)	9 (45.00%)	12 (60.00%)	<0.001 #, ##	0.22	0.23

# T0 vs. T1, ## T0 vs. T2. MMP-9—metalloproteinase-9, TIMP-1—tissue inhibitor of metalloproteinase-1, CRP—C-reactive protein, fCal—fecal calprotectin, PUCAI—Pediatric activity index, PCDAI—pediatric Crohn’s disease activity index, NA—not applicable.

## Data Availability

The original contributions presented in this study are included in the article. Further inquiries can be directed to the corresponding author.

## Supplementary Materials

The following supporting information can be downloaded at: https://www.mdpi.com/article/10.3390/ijms27209162/s1.

## References

[1] Levine A., Koletzko S., Turner D., Escher J.C., Cucchiara S., de Ridder L., Kolho K., Veres G., Russell R.K., Paerregaard A. (2014). ESPGHAN revised porto criteria for the diagnosis of inflammatory bowel disease in children and adolescents. J. Pediatr. Gastroenterol. Nutr..

[2] Diez-Martin E., Hernandez-Suarez L., Muñoz-Villafranca C., Martin-Souto L., Astigarraga E., Ramirez-Garcia A., Barreda-Gómez G. (2024). Inflammatory Bowel Disease: A Comprehensive Analysis of Molecular Bases, Predictive Biomarkers, Diagnostic Methods, and Therapeutic Options. Int. J. Mol. Sci..

[3] Granlund A., Flatberg A., Østvik A.E., Drozdov I., Gustafsson B.I., Kidd M., Beisvag V., Torp S.H., Waldum H.L., Martinsen T.C. (2013). Whole genome gene expression meta-analysis of inflammatory bowel disease colon mucosa demonstrates lack of major differences between Crohn’s disease and ulcerative colitis. PLoS ONE.

[4] Bouma G., Strober W. (2003). The immunological and genetic basis of inflammatory bowel disease. Nat. Rev. Immunol..

[5] Zdanowicz K., Pietrowska K., Lebensztejn D.M., Ciborowski M., Godzien J., Kretowski A., Daniluk J., Daniluk U. (2025). Evaluation of microbiome composition combined with serum untargeted metabolomic profiling in newly diagnosed children with inflammatory bowel disease. Arch. Med. Sci..

[6] Jakobsen C., Bartek J., Wewer V., Vind I., Munkholm P., Groen R., Paerregaard A. (2011). Differences in phenotype and disease course in adult and paediatric inflammatory bowel disease—A population-based study. Aliment. Pharmacol. Ther..

[7] Zdanowicz K., Czajkowska A., Rycyk A., Lebensztejn D.M., Daniluk U. (2025). Early and simple laboratory markers of poor outcome in children with inflammatory bowel disease. Arch. Med. Sci..

[8] Marônek M., Marafini I., Gardlík R., Link R., Troncone E., Monteleone G. (2021). Metalloproteinases in Inflammatory Bowel Diseases. J. Inflamm. Res..

[9] Mei K., Chen Z., Wang Q., Luo Y., Huang Y., Wang B., Gu R. (2022). The role of intestinal immune cells and matrix metalloproteinases in inflammatory bowel disease. Front. Immunol..

[10] Baum S., Hamedi K., Loftus C., Loftus G., Zhou E.R., Arce S. (2025). From Cytokines to Biomarkers: Mapping the Immunopathology of Inflammatory Bowel Disease. Cells.

[11] Schoeps B., Frädrich J., Krüger A. (2023). Cut loose TIMP-1: An emerging cytokine in inflammation. Trends Cell Biol..

[12] Sosna B., Aebisher D., Myśliwiec A., Dynarowicz K., Bartusik-Aebisher D., Oleś P., Cieślar G., Kawczyk-Krupka A. (2023). Selected Cytokines and Metalloproteinases in Inflammatory Bowel Disease. Int. J. Mol. Sci..

[13] de Paulo C.B., Miglino M.A., Castelucci P. (2024). Perspectives on the extracellular matrix in inflammatory bowel disease and bowel decellularization protocols. World J. Exp. Med..

[14] Daniluk U., Daniluk J., Guzinska-Ustymowicz K., Pryczynicz A., Lebensztejn D. (2020). Usefulness of metalloproteinase-9 and tissue inhibitor of metalloproteinase-1 in clinical characterisation of children with newly diagnosed Crohn’s disease. J. Paediatr. Child. Health.

[15] Czajkowska A., Guzinska-Ustymowicz K., Pryczynicz A., Lebensztejn D., Daniluk U. (2022). Are Matrix Metalloproteinase-9 and Tissue Inhibitor of Metalloproteinase-1 Useful as Markers in Diagnostic Management of Children with Newly Diagnosed Ulcerative Colitis?. J. Clin. Med..

[16] Sato T., Ito A., Mori Y. (1990). Interleukin 6 enhances the production of tissue inhibitor of metalloproteinases (TIMP) but not that of matrix metalloproteinases by human fibroblasts. Biochem. Biophys. Res. Commun..

[17] Takeshita S., Tokutomi T., Kawase H., Nakatani K., Tsujimoto H., Kawamura Y., Sekine I. (2001). Elevated serum levels of matrix metalloproteinase-9 (MMP-9) in Kawasaki disease. Clin. Exp. Immunol..

[18] Klimiuk P.A., Sierakowski S., Domyslawska I., Chwiecko J. (2004). Effect of repeated infliximab therapy on serum matrix metalloproteinases and tissue inhibitors of metalloproteinases in patients with rheumatoid arthritis. J. Rheumatol..

[19] Mäkitalo L., Rintamäki H., Tervahartiala T., Sorsa T., Kolho K.L. (2012). Serum MMPs 7-9 and their inhibitors during glucocorticoid and anti-TNF-α therapy in pediatric inflammatory bowel disease. Scand. J. Gastroenterol..

[20] Gao Q., Meijer M.J., Schlüter U.G., van Hogezand R.A., van der Zon J.M., van den Berg M., van Duijn W., Lamers C.B., Verspaget H.W. (2007). Infliximab treatment influences the serological expression of matrix metalloproteinase (MMP)-2 and -9 in Crohn’s disease. Inflamm. Bowel Dis..

[21] de Bruyn M., Arijs I., De Hertogh G., Ferrante M., Van Assche G., Rutgeerts P., Vermeire S., Opdenakker G. (2015). Serum Neutrophil Gelatinase B-associated Lipocalin and Matrix Metalloproteinase-9 Complex as a Surrogate Marker for Mucosal Healing in Patients with Crohn’s Disease. J. Crohns Colitis.

[22] Carbone F., Bodini G., Brunacci M., Bonaventura A., Vecchiè A., Liberale L., Crespi M., Baldissarro I., Dallegri F., Savarino V. (2018). Reduction in TIMP-2 serum levels predicts remission of inflammatory bowel diseases. Eur. J. Clin. Investig..

[23] Zdanowicz K., Maciejczyk M., Jamiołkowski J., Lebensztejn D.M., Daniluk U. (2025). Serum cytokine inflammatory profile in children with ulcerative colitis during 6-month follow-up. Sci. Rep..

[24] Turner D., Otley A.R., Mack D., Hyams J., de Bruijne J., Uusoue K., Walters T.D., Zachos M., Mamula P., Beaton D.E. (2007). Development, validation, and evaluation of a pediatric ulcerative colitis activity index: A prospective multicenter study. Gastroenterology.

[25] Turner D., Griffiths A.M., Walters T.D., Seah T., Markowitz J., Pfefferkorn M., Keljo D., Otley A., LeLeiko N.S., Mack D. (2010). Appraisal of the pediatric Crohn’s disease activity index on four prospectively collected datasets: Recommended cutoff values and clinimetric properties. Am. J. Gastroenterol..

[26] Levine A., Griffiths A., Markowitz J., Wilson D.C., Turner D., Russell R.K., Fell J., Ruemmele F.M., Walters T., Sherlock M. (2011). Pediatric modification of the Montreal classification for inflammatory bowel disease: The Paris classification. Inflamm. Bowel Dis..

[27] Daperno M., D’Haens G., Van Assche G., Baert F., Bulois P., Maunoury V., Sostegni R., Rocca R., Pera A., Gevers A. (2004). Development and validation of a new, simplified endoscopic activity score for Crohn’s disease: The SES-CD. Gastrointest. Endosc..

[28] Turner D., Ruemmele F.M., Orlanski-Meyer E., Griffiths A.M., De Carpi J.M., Bronsky J., Veres G., Aloi M., Strisciuglio C., Braegger C.P. (2018). Management of Paediatric Ulcerative Colitis, Part 1: Ambulatory Care—An Evidence-based Guideline from European Crohn’s and Colitis Organization and European Society of Paediatric Gastroenterology, Hepatology and Nutrition. J. Pediatr. Gastroenterol. Nutr..

[29] Turner D., Ruemmele F.M., Orlanski-Meyer E., Griffiths A.M., De Carpi J.M., Bronsky J., Veres G., Aloi M., Strisciuglio C., Braegger C.P. (2018). Management of Paediatric Ulcerative Colitis, Part 2: Acute Severe Colitis—An Evidence-based Consensus Guideline from the European Crohn’s and Colitis Organization and the European Society of Paediatric Gastroenterology, Hepatology and Nutrition. J. Pediatr. Gastroenterol. Nutr..

[30] van Rheenen P.F., Aloi M., Assa A., Bronsky J., Escher J.C., Fagerberg U.L., Gasparetto M., Gerasimidis K., Griffiths A., Henderson P. (2021). The Medical Management of Paediatric Crohn’s Disease: An ECCO-ESPGHAN Guideline Update. J. Crohn’s Colitis.

[31] Ruemmele F.M., Veres G., Kolho K.L., Griffiths A., Levine A., Escher J.C., Amil Dias J., Barabino A., Braegger C.P., Bronsky J. (2014). Consensus guidelines of ECCO/ESPGHAN on the medical management of pediatric Crohn’s disease. J. Crohn’s Colitis.

[32] Koninckx C.R., Donat E., Benninga M.A., Broekaert I.J., Gottrand F., Kolho K.L., Lionetti P., Miele E., Orel R., Papadopoulou A. (2021). The Use of Fecal Calprotectin Testing in Paediatric Disorders: A Position Paper of the European Society for Paediatric Gastroenterology and Nutrition Gastroenterology Committee. J. Pediatr. Gastroenterol. Nutr..

[33] Kristensen V., Røseth A., Ahmad T., Skar V., Moum B. (2017). Fecal Calprotectin: A Reliable Predictor of Mucosal Healing after Treatment for Active Ulcerative Colitis. Gastroenterol. Res. Pract..

