# Supplementary material for: Changes in Serum MMP-9 and TIMP-1 Concentrations in Children with Inflammatory Bowel Disease over a Six-Month Observation Period
Source: Int J Mol Sci. 2026 Oct 15;27(20):9162. doi: 10.3390/ijms27209162 (PMC13704964; doi:10.3390/ijms27209162)
Supplement: Supplementary file 1 [file ijms-27-09162-s001.zip › ijms-4615942-supplementary.pdf]

Table S1. Correlation analysis between MMP-9, TIMP-1 concentrations and the MMP-9/TIMP-1 ratio at baseline (T0). Correlation coefficients (r) and p-values are presented.

	MMP-9 (T0)	TIMP-1 (T0)	MMP-9/TIMP-1 ratio (T0)
IBD			
CRP (T0)	R=0.08; p=0.55	R=0.24, p=0.07	R=-0.12, p=0.35
fCal (T0)	R=-0.09, p=0.49	R=-0.18, p=0.18	R=0.07, p=0.60
PUCAI (T0)	R=-0.15, p=0.26	R=0.02, p=0.86	R=-0.15, p=0.26
UC			
CRP (T0)	R=0.19, p=0.25	R=0.14, p=0.40	R=0.13, p=0.54
fCal (T0)	R=-0.01, p=0.98	R=-0.11, p=0.50	R=0.27, p=0.20
PUCAI (T0)	R=-0.16, p=0.32	R=-0.01, p=0.95	R=-0.05, p=0.82
CD			
CRP (T0)	R=0.29, p=0.22	R=0.35, p=0.15	R=0.06, p=0.81
fCal (T0)	R=-0.35, p=0.15	R=-0.45, p=0.06	R=0.12, p=0.62
PCDAI (T0)	R=-0.24, p=0.32	R=0.28, p=0.24	R=-0.36, p=0.12

MMP-9 metalloproteinase-9, TIMP-1—tissue inhibitor of metalloproteinase-1, CRP—C-reactive protein, fCal—fecal calprotectin, PUCAI-Pediatric activity index, PCDAI- pediatric Crohn's disease activity index

Table S2a Correlation analysis between MMP-9, TIMP-1 concentrations and the MMP-9/TIMP-1 ratio after 3 months of follow-up (T1). Correlation coefficients (R) and p-values are presented.

	MMP-9 (T1)	TIMP-1 (T1)	MMP-9/TIMP-1 ratio (T1)
IBD			
CRP (T1)	R=0.09, p=0.50	R=0.39, p=0.002	R=-0.19, p=0.14
fCal (T1)	R=0.25, p=0.07	R=0.03, p=0.83	R=0.23, p=0.009
PUCAI/PCDAI (T1)	R=-0.01, p=0.99	R=0.18, p=0.19	R=-0.12, p=0.28
UC			
CRP (T1)	R=0.29, p=0.07	R=0.28, p=0.08	R=0.03, p=0.90
fCal (T1)	R=0.17, p=0.31	R=-0.05, p=0.76	R=0.28, p=0.20
PUCAI (T1)	R=001, p=0.98	R=0.10, p=0.52	R=0.02, p=0.92
CD			
CRP (T1)	R=-0.14, p=0.58	R=0.60, p=0.007	R=-0.49, p=0.03
fCal (T1)	R=0.47, p=0.05	R=0.31, p=0.21	R=0.19, p=0.46
PCDAI (T1)	R=-0.01, p=0.99	R=0.24, p=0.32	R=-0.24, p=0.32

MMP-9 metalloproteinase-9, TIMP-1—tissue inhibitor of metalloproteinase-1, CRP—C-reactive protein, fCal—fecal calprotectin, PUCAI-Pediatric activity index, PCDAI- pediatric Crohn's disease activity index

Table S2b Correlation analysis between MMP-9, TIMP-1 concentrations and the MMP-9/TIMP-1 ratio after 6 months of follow-up (T2). Correlation coefficients (R) and p-values are presented.

	MMP-9 (T2)	TIMP-1 (T2)	MMP-9/TIMP-1 ratio (T2)
IBD			
CRP (T2)	R=0.09, p=0.50	R=0.17, p=0.20	R=-0.14, p=0.30
fCal (T2)	R=0.13, p=0.32	R=-0.13, p=0.34	R=0.26, p=0.05
PUCAI/PCDAI (T2)	R=-0.01, p=0.97	R=-0.04, p=0.75	R=0.07, p=0.61
UC			
CRP (T2)	R=0.18, p=0.28	R=0.04, p=0.83	R=0.16, p=0.51
fCal (T2)	R=0.01, p=0.93	R=-0.25, p=0.17	R=0.49, p=0.03
PUCAI (T2)	R=-0.01, p=0.95	R=-0.05, p=0.75	R=0.14, p=0.57
CD			

CRP (T2)	R=0.04, p=0.87	R=0.51, p=0.03	R=-0.17, p=0.48
fCal (T2)	R=0.40, p=0.10	R=0.61, p=0.007	R=0.21, p=0.40
PCDAI (T2)	R=-0.01, p=0.97	R=0.16, p=0.52	R=-0.01, p=0.97

MMP-9 metalloproteinase-9, TIMP-1—tissue inhibitor of metalloproteinase-1, fCal—fecal calprotectin, PUCAI-Pediatric activity index, PCDAI- pediatric Crohn's disease activity index

Supplementary Material - Methods

The assay detection ranges were 0.156–10 ng/mL for MMP-9 and 1.56–100 ng/mL for TIMP-1. Serum samples were diluted 1:100 for MMP-9 and 1:10 for TIMP-1, and the respective dilution factors were applied to calculate the final serum concentrations. The manufacturers report intra-assay coefficients of variation of <10% and inter-assay coefficients of variation of <12% for both assays. A total of 204 serum samples (177 samples from patients with IBD and 27 control samples) were analyzed using 96-well ELISA plates, requiring 8 plates for each analyte. Seven wells were used for the calibration standards and one well for the blank, leaving 88 wells for samples. As each serum sample was analyzed in triplicate, up to 29 individual samples could be analyzed per plate. Samples from both the IBD and control groups, as well as from different time points (T0, T1 and T2), were distributed across the plates to minimize potential plate-related variability. Laboratory personnel performing the assays were blinded to the clinical group and time point. Each serum sample was analyzed in triplicate for both MMP-9 and TIMP-1. The mean of the three measurements was used for further statistical analysis. All measured values were within the calibration range of the respective assays after application of the appropriate sample dilution factors.
